# Computational frameworks for automated detection and quantification of paroxysmal sympathetic hyperactivity among traumatic brain injury patients

**DOI:** 10.1371/journal.pone.0344088

**Published:** 2026-03-03

**Authors:** Xiangxiang Kong, Lujie Karen Chen, Sancharee Hom Chowdhurry, Ryan B. Felix, Shiming Yang, Peter Hu, Neeraj Badjatia, Jamie Erin Podell

**Affiliations:** 1 University of Maryland, Baltimore, United States of America; 2 University of Maryland, School of Medicine, United States of America; University of Washington, UNITED STATES OF AMERICA

## Abstract

Paroxysmal sympathetic hyperactivity (PSH) is a syndrome that occurs in a large subset of critically ill traumatic brain injury (TBI) patients and is associated with complications and poor recovery. PSH is defined by recurrent episodic vital sign elevations in the appropriate clinical context. However, standard diagnostic criteria rely heavily on subjective judgment, leading to challenges and delays in recognition, monitoring, and management. The objective of this study was to develop automated PSH detection and quantification tools that exclusively utilize objective bedside continuous vital sign data. Using a cohort of 221 critically ill acute TBI patients with at least 14 days of continuous physiologic data (of which 107 were clinically diagnosed with PSH) we developed a high-resolution clinical feature scale based on established PSH-Assessment Measure criteria and two artificial intelligence-based episode detection models including an expert system approach and a machine learning model approach, using a clinician-annotated case example as ground truth. For the episode detection methods, PSH was quantified as the number, duration, and overall temporal burden of detected episodes. To evaluate performance, we compared quantifications across PSH cases and controls and explored precision and recall. All three methods demonstrated initial face validity to delineate PSH cases from non-PSH TBI controls. Future optimization and implementation of the described computational frameworks with real-time patient data could improve the standard monitoring and management of this challenging clinical syndrome.

## Introduction

Paroxysmal Sympathetic Hyperactivity (PSH) is a clinically important manifestation of autonomic dysfunction that occurs in up to one third of critically ill patients after traumatic brain injury (TBI) [1–5]. Recurrent, sympathetically-mediated episodes of tachycardia, hypertension, tachypnea, hyperthermia, and motor posturing define PSH. Its occurrence has been associated with worse outcomes and systemic complications after TBI, suggesting that it may represent a modifiable risk factor for poor outcomes after TBI via a number of potential mechanisms. For example. sympathetic hyperactivity after TBI has been associated with a pro-inflammatory state, coagulopathy, and endothelial dysfunction [6]. In addition, high temperatures and extreme vital sign fluctuations may contribute to secondary inflammatory, ischemic, and hemorrhagic brain injury, cardiovascular complications, acute kidney injury, etc. [3]. Further, management strategies for PSH rely on multimodal abortive and preventative symptom-suppressing medications including sedatives [7] that can prolong coma, immobility, and ventilator dependence, further increasing the risk of healthcare associated complications and poor recovery.

Effective management of PSH requires prompt recognition and careful observation of symptom severity over time. Early and accurate detection may facilitate proactive management strategies to avoid PSH-related complications and thereby improve recovery from TBI. Unfortunately, clinical recognition of PSH is difficult and often delayed. Automated PSH detection and quantification tools are currently lacking, but if implemented could support decision making around this challenging clinical syndrome [8].

In recent years, the expert consensus-based PSH-Assessment Measure (PSH-AM) has emerged as the research and clinical standard for determining the diagnostic likelihood and clinical severity of PSH [1,2,9–11] (Table 1). However, several limitations restrict its effectiveness as a real-time detection and monitoring tool [8]. First, calculating the PSH-AM score requires manual extraction of daily vital signs (VS) from the Electronic Health Record (EHR) and querying bedside providers for specific information not typically documented in the EHR, such as the severity of sweating and posturing, the frequency of episodes, simultaneity of features, and any perceived triggers. This process is both inefficient and also limited by data ascertainment. For example, hourly VS documented in the EHR may fail to capture clinically relevant extreme episodic VS derangements lasting only 15 minutes. Second, the threshold-based clinical feature scale (CFS) score fails to fully capture the episodic nature of PSH, a core feature of the syndrome.

**Table 1:** Paroxysmal Sympathetic Hyperactivity-Assessment Measure (PSH-AM).

Clinical feature scale (CFS)					
	0	1	2	3	Score
Heart rate	<100	100-119	120-139	≥140	
Respiratory rate	<18	18-23	24-29	≥30	
Systolic blood pressure	<140	140-159	160-179	≥180	
Temperature	<37	37-37.9	38-38.9	≥39	
Sweating	Nil	Mild	Moderate	Severe	
Posturing	Nil	Mild	Moderate	Severe	
			**CFS subtotal**		
**Diagnosis likelihood tool (DLT)**					
Clinical features occur simultaneously					
Episodes are paroxysmal in nature					
Sympathetic over-reactivity to normally non-painful stimuli					
Features persist ≥3 consecutive days					
Features persist ≥2 weeks					
Features persist despite treatment of alternative differential diagnoses					
Medication administered to decrease sympathetic features					
≥2 episodes daily					
Absence of parasympathetic features during episodes					
Absence of other presumed cause of features					
Antecedent acquired brain injury					
			**DLT subtotal**		
**Combined total (CFS + DLT)**			Unlikely	<8	
			Possible	8-16	
			Probable	≥17	

Overall, PSH episode detection has not been well-operationalized in real-world clinical settings to support real-time patient monitoring. While bedside continuous VS monitoring is ubiquitous in critical care units where PSH is typically first encountered, there is no automatic physiologic detection and monitoring tool for PSH. Further, despite it being defined by its physiologic derangements, no studies to date have aimed to model PSH based exclusively on physiological data [8].

In this study, we explore computational frameworks for modeling and detecting PSH using densely sampled physiological VS data collected from bedside monitors. We first derive a simple automated high-resolution modified CFS tool that overcomes the low sampling frequency limitation of the standard EHR-based CFS. Our next focus is on the feasibility of automatically detecting PSH *episodes* in real time. We hypothesize that computational models, supported by advanced data collection and processing infrastructure, can generate outputs that distinguish TBI patients who were ultimately clinically diagnosed with PSH from those who were not. Validating this approach could open the avenues for real-time monitoring and goal-directed management of PSH in neurocritical care settings.

## Methods

### Patient cohort

In this study, we used a cohort of critically ill adult TBI patients (N = 221) admitted to R Adams Cowley Shock Trauma Center of University of Maryland Medical Center between January 2016 and July 2018. The inclusion criteria were head Abbreviated Injury Scale (AIS) > 0, ICU length of stay of at least three days and hospital length of stay of at least 14 days. Patients with mild uncomplicated TBI (GCS < 12 and negative head CT) and spinal cord injuries were excluded. The cohort was separated into two groups: 107 case patients who were treated with propranolol or bromocriptine for at least three days for suspected PSH, with indication further confirmed via medical record review, and 114 control patients who were never treated with those medications. The rationale for inclusion/exclusion criteria and PSH case definitions have been discussed in previous work [1,12–14]. We calculated summary statistics to describe demographic, injury, radiographic, and clinical characteristics of our patient cohort.

Multivariate VS time series data was recorded from bedside monitors as part of standard clinical care for all patients. Data trends including heart rate (HR), respiratory rate (RR), temperature (TMP), peripheral oxygen saturation (SpO2) and systolic blood pressure (SBP) were collected from photoplethysmography (PPG), electrocardiography (ECG), arterial blood pressure, noninvasive blood pressure, and temperature monitors with a sampling frequency of 0.5 Hz. In total, we had approximately 3000 patient days of multivariate high-resolution VS time series data.

Electronic health record data was accessed and linked to recorded physiologic data between January 30, 2019 and December 30, 2019. After using unique identifiers to link data from different sources, personally identifiable information was removed from all data prior to analysis to ensure privacy and that individual patients cannot be identified. The study was approved by the Institutional Review Board of the University of Maryland, Baltimore, which waived the need for informed consent.

### PSH quantification

We used the aforementioned VS time series data to develop automatic PSH quantification tools. First, we derived a high-resolution CFS (hrCFS) to quantify the severity of VS derangements in keeping with the threshold-based scoring system of the PSH-AM’s CFS. Next, we explored methods that identify and quantify PSH *episodes*, where the paroxysmal nature of physiologic changes is the central focus.

### High-resolution CFS (hrCFS) quantification methods

In keeping with parameters set by the PSH-AM, we developed a high-resolution CFS (hrCFS) derived from continuously collected VS data. The hrCFS is automatically calculated across time series data based on VS thresholds from the CFS subscore of the PSH-AM (Table 1). Traditionally, CFS scores are calculated as a daily maximum based on clinical observations of episodes rather than continuous physiological data(2). Our metric is termed “high-resolution,” as it calculates an instantaneous modified CFS score at the highest resolution of VS data recordings (e.g., 0.5 Hz). This increases the sensitivity to detect true maximum values during any given time frame. It is modified from the traditional CFS because it excludes sweating, posturing, and temperature components, which cannot be reliably derived at high resolution from our VS data. The range of hrCFS is therefore 0–9.

**Fig 1:**
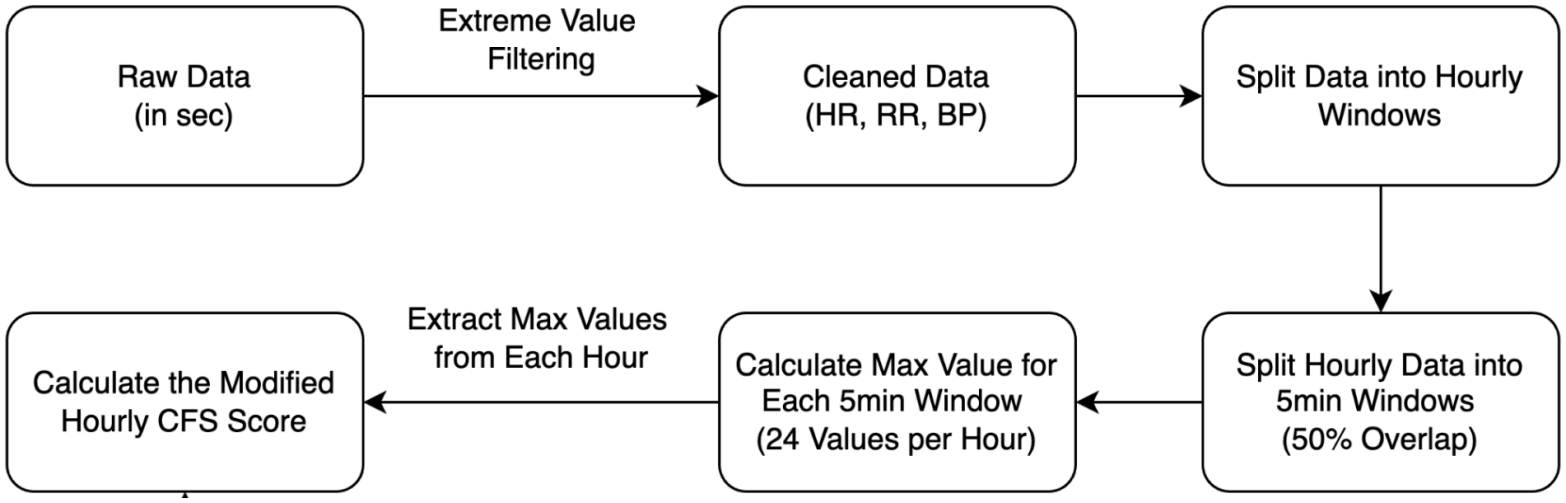
High resolution clinical feature scale (hrCFS). Method illustration for calculation of hourly high-resolution clinical feature scale (hrCFS) based on 0.5 Hz vital sign trend data.

An overview of our methods for calculating the hrCFS is illustrated in Fig 1. To ensure data quality, an extreme value filtering approach was applied to heart rate (HR), respiratory rate (RR) and systolic blood pressure (SBP) measurements to remove artifacts. For instance, for adult patients, heart rate values were considered within a normal reference range of 30–250 beats per minute. After pre-processing, the cleaned data was segmented into hourly intervals. Each hourly interval was further divided into 5-minute rolling windows with 50% overlap (2.5-minute step size) to capture temporal patterns at a finer granularity. From each 5-minute window, the maximum value of each VS (HR, RR, and SBP) was extracted, resulting in 24 values per hour. Finally, the maximum value of each VS was selected to represent the hourly summary. The extracted hourly values of VS (HR, RR, and BP) were then quantified on a 0–3 scale, following the PSH-AM criteria (Table 1). The quantified values were summed to compute the composite hrCFS score.

### PSH episode detection quantification methods

Our proof of concept PSH episode detection and quantification methods involve expert annotation of physiologic data to inform models that automatically label physiologic data into episodes, which may then be summarized as a PSH burden score. These steps are described below.

### Episode annotation (ground truth)

A neurocritical care clinician (JEP) visualized and annotated the high resolution VS time series data from one randomly selected TBI patient with PSH using an existing open-source data visualization and annotation tool, Auton Universal Viewer (AUViewer, https://auviewer.readthedocs.io/en/latest/). The clinician marked episodes that aligned with descriptions of PSH in the literature, with simultaneous and paroxysmal elevations in heart rate, respiratory rate, systolic blood pressure, and temperature that were brief (typically lasting around 15 minutes but ranging from 2 minutes to 2 hours), recurrent (often with at least three episodes per day), and characterized by a relatively abrupt onset and offset [2,15,16]. The clinician marked the onset and offset of events based on heart rate or respiratory rate data obtained from either the photoplethysmography (PPG) or electrocardiography (ECG) signals. While simultaneous elevations in blood pressure and temperature supported the recognition of discrete PSH events, these features were not used to label events due to their lower temporal resolution and higher incidence of missing data. In total, the training data provided by this case example included 180 episodes identified by the expert clinician over the course of 622.8 hours (26 days) of recorded data. Episode mean duration was 42 minutes and standard deviation was 16 minutes; duration ranged from 3 to 206 minutes.

### Episode detection tool derivation

We next explored two artificial intelligence (AI) methods utilizing annotated data to automatically detect PSH episodes from high resolution VS data: a manually derived expert system (ES) based approach and a machine learning model approach [17–19]. The ES method involved manually crafting rules based on statistical features extracted from the clinician-annotated episodes described above, while the model-based approach used a supervised machine learning method Support Vector Machine (SVM) model to derive rules by comparing characteristics of physiological signals within annotated PSH episodes to baseline inter-episode signals. All rules were derived from and applied to heart rate data, which was high quality and continuous in our dataset.

**Fig 2:**
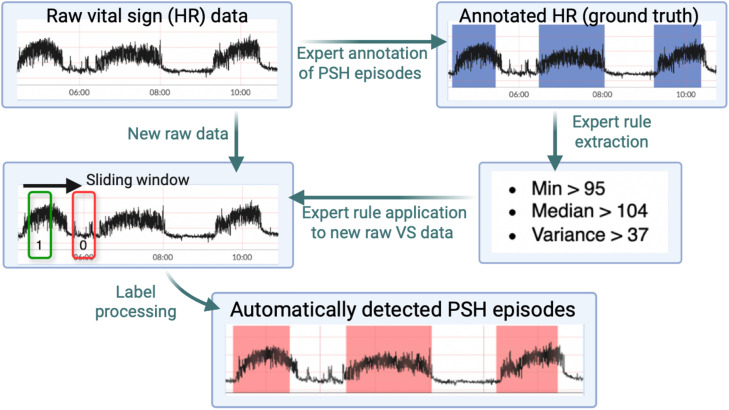
Expert system-based episode detection method. Flow diagram illustrating the derivation process for the expert system (ES) method of detecting paroxysmal sympathetic hyperactivity (PSH) episodes based on an expert clinician’s annotations of continuous vital sign (VS) data from a patient with PSH. Annotation is performed by the expert clinician and rule extraction by the data analyst.

For the manual rule-based method (Fig 2), the primary data analyst (XK) noted that clinician-annotated episodes were characterized by a sudden increase in HR followed by a stably elevated heart rate with high variance that persists for minutes to hours. Based on this, three parameterized rules were constructed: HR minimum > *a*, HR median > *b*, and HR variance > *c*. The parameters (*a*, *b*, and *c*) were identified using grid search to optimize precision and recall with clinical annotation as the ground truth. The final rule set based on single patient training data was HR minimum > 95, HR median > 104, and HR variance > 37.

We then apply these rules to raw HR time series data from all included TBI patients in the dataset. For each patient’s data, we apply the rules using a series of sliding windows of 10-minute width with a step size of 2 seconds, resulting in a series of binary indicators for each sliding window. Windows that satisfy at least two of the three rules are classified as positive, while the rest are classified as negative. We then assigned all data points within a given window the same label (i.e., all data points within a positive window were labeled positive, and vice versa). In cases where data points were assigned conflicting indicators, we used the maximum of those binary indicators as the final label for each data point. This process produced a series of binary indicators at the data point level. To further process these binary indicators into PSH episodes, we merged consecutive series of positive indicators into PSH episodes. Since it is unlikely for two consecutive episodes to have a short interval between them, we also merged episodes with a break of less than 1 minute.

**Fig 3:**
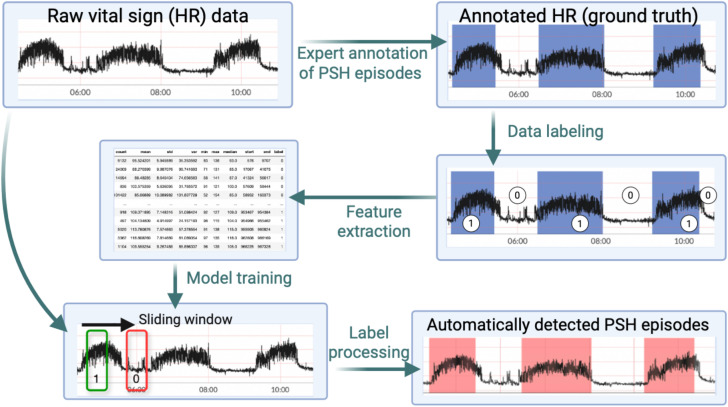
Machine learning model-based episode detection method. Flowchart illustrating the derivation process for the support vector machine (SVM) model-based method of detecting paroxysmal sympathetic hyperactivity (PSH) episodes using an expert clinician’s annotations of continuous vital sign (VS) data from a patient with PSH. Annotation is performed by the expert clinician and rule extraction by the data analyst.

The machine learning model-based episode detection method is illustrated in Fig 3. After labeling HR data from the clinician-annotated episodes as positive and inter-episode baseline intervals as negative from the single case example, we extracted statistical features (mean, standard deviation, minimum, maximum, and median). These feature sets were derived from rolling windows of size 10 minutes with a step size of 2.5 minutes (i.e., 75% overlap). The reason for choosing a much larger step size compared to the rule-based method is to limit the redundancy for the training dataset. The entire feature set, along with the labels, was then used to train the SVM classification model, with radial basis function kernel [19]. We then applied the SVM model to each patient’s raw HR data by labeling rolling windows either negative (baseline/non-anomalous) or positive (episode/anomalous). PSH episodes were derived from the series of binary indicators using a similar approach to the rule-based method.

### Episode detection tool performance evaluation

In this section, we use two approaches to evaluate the performance of the automatic episode detection methods described above. The first approach aims to validate the approaches by applying the models to our full patient cohort and comparing the aggregate “burden” of PSH episodes between PSH-positive and PSH-negative patients. The second approach directly measures the precision and recall of the automatic annotation compared to the human annotation for the single case example.

### PSH episode detection performance evaluation: PSH burden

We define the burden score as the temporal density of PSH episodes, quantifying the proportion of time that a patient experiences PSH episode(s) within a period of time. Suppose there are *k* episodes within a given time frame, *T*; the burden score, *B*, is calculated using the following equation:


$$B\;=\;\frac{\text{1}}{T}\sum_{n=\text{1}}^{k}duration\;of\;episode\;\epsilon_{n}$$

where, $\epsilon_{n}$ represents the PSH episode duration. For example, if a patient has a cumulative 2 hours of HR data labeled as PSH episodes over a 4-hour time frame, the burden of PSH episodes within 4 hours is 0.5. The same score could be achieved by multiple short episodes or a single long episode.

As a face validity check of the PSH episode detection-derived burden scores, we compared the burden score trends across clinically diagnosed PSH cases and control TBI patients. For each patient, the PSH episode burden was calculated over consecutive 12-hour periods from time zero (start of VS recording, which occurs on admission at the time the patient arrives in the trauma resuscitation unit). The mean burden score and 95% confidence interval are plotted over time comparing PSH cases to controls. Plots are created for both episode detection method-based burden scores and compared to trends derived from the hrCFS. Additionally, for each episode detection method, considering the first 14 days of hospitalization for each patient, we summarize the total number of episodes, mean duration of episodes, and overall PSH episode burden, comparing these values across PSH cases and controls.

### PSH episode detection performance evaluation: precision and recall

Precision and recall are common performance measures used to evaluate the accuracy of machine learning models by comparing their outputs with the ground truth. In our application, however, detected episodes are described as ranges or durations rather than individual data points. Consequently, when comparing with ground truth annotations, there may be partial overlaps between detected and ground truth annotated episodes. To properly account for this, we compute modified precision and recall, as proposed by Tatbul et al. [20]. The formulas for computing modified precision and recall are as follows:

Precision score:


$$Precision\;(R,\;P)\;=\frac{\sum_{i=\text{1}}^{N_{P}}Precision\;(R,\;P_{i})}{N_{P}}\;$$

Recall score:


$$Recall\;(R,\;P)\;=\;\frac{\sum_{i=\;\text{1}}^{N_{r}}Recall\;(R_{i}\;,\;P)}{N_{r}}$$

Where *R* is the set of annotated episodes and *P* is the set of automatically detected episodes, *N*_*r*_ and *N*_*p*_ are the number of annotated and detected episodes, respectively.

### Author generated code

Author-generated code is freely available at: https://github.com/seankong88/PSH_episode_analysis

## Results

### Included patient characteristics

Our cohort included 221 critically ill TBI patients described in Table 2.

**Table 2:** Included patient characteristics.

	All	PSH (+)	PSH (-)
Included patients, n	221	107	114
**Demographics**			
Age, years	44 (19)	37 (15)	51 (19)
BMI	27 (11)	27 (5)	27 (14)
Sex, n (%)			
- Male	175 (79)	92 (86)	83 (73)
Race, n (%)			
- White	115 (52)	49 (46)	66 (58)
- Black	80 (36)	42 (39)	38 (33)
- Others	26 (12)	16 (15)	10 (9)
Ethnicity, n (%)			
- Hispanic/Latino	27 (12)	15 (14)	12 (11)
**Injury Characteristics**			
Type, n (%)			
- Non-penetrating	202 (91)	99 (93)	103 (90)
- Penetrating	19 (9)	8 (7)	11 (10)
Injury Severity Score	26 [19, 34]	26 [21, 33]	26 [18, 34]
Head AIS	4 [3, 5]	4 [3, 5]	4 [3, 5]
Trauma injury severity score	0.75 [0.48, 0.90]	0.71 [0.45, 0.87]	0.80 [0.49, 0.93]
**Admission Clinical Characteristics**			
GCS score on ICU admission			
- Eye	1 [1, 3]	1 [1, 2]	1 [1, 3]
- Motor	4 [2, 5]	4 [3, 5]	5 [1, 6]
- Verbal	1 [1, 1]	1 [1, 1]	1 [1, 1]
- Total	7 [4, 9]	7 [5, 8]	7 [4, 10]
Following commands on ICU admission, n (%)	49 (22)	17 (16)	32 (28)
Had Brain Surgery, n (%)	68 (31)	31 (29)	37 (32)
Intracranial Pressure Monitor Placed, n (%)	120 (54)	80 (75)	40 (35)
**Initial CT Radiographic Characteristics**			
Marshall Score	2 [2, 4]	2 [2, 4]	2 [2, 4]
Total Rotterdam CT score	3 [3, 4]	3 [3, 4]	3 [3, 4]
Basal cistern compression, n (%)			
- Partial	52 (24)	22 (21)	30 (26)
- Complete	22 (10)	15 (14)	7 (6)
Midline shift of> or = 5 mm, n (%)	59 (27)	26 (24)	33 (29)
Absence of epidural hemorrhage, n(%)	198 (90)	96 (90)	102 (89)
Presence of Intraventricular or subarachnoid hemorrhage, n (%)	163 (74)	86 (80)	77 (68)
Contusion, n (%)	105 (48)	46 (43)	59 (52)
Intraventricular hemorrhage, n (%)	43 (19)	22 (21)	21 (18)
Hydrocephalus, n (%)	21 (10)	12 (11)	9 (8)
Extra-axial hemorrhage, n (%)	142 (64)	61 (57)	81 (71)
Diffuse axonal injury, n (%)	36 (16)	27 (25)	9 (8)
**Hospital Discharge Outcome**			
Survived, n (%)	207 (94)	102 (95)	105 (92)
GCS at the hospital discharge			
- Eye	4 [4, 4]	4 [4, 4]	4 [4, 4]
- Motor	6 [5, 6]	6 [5, 6]	6 [6, 6]
- Verbal	3 [1, 5]	1 [1, 4]	4 [1, 5]
- Total	13 [10, 15]	11 [10, 14]	14 [11, 15]
Following commands at hospital discharge, n (%)	160 (72)	70 (65)	90 (79)
Hospital LOS, days	29 (14)	30 (15)	27 (13)
ICU LOS, days	20 (10)	20 (9)	19 (12)
Days on Ventilation	14 (9)	15 (7)	13 (11)
DLT	4 [2, 8]	8 [8, 9]	2 [1, 3]

Continuous variables are expressed as mean (SD). Ordinal or non-normally distributed continuous variables are expressed as median (25th, 75th percentile). Categorical variables are expressed as count (percentage). AIS: Abbreviated Injury Score; BMI: Body Mass Index; DLT: Diagnostic Likelihood Tool score; GCS: Glasgow Coma Scale; ICU: Intensive Care Unit; LOS: Length of Stay.

### Data underlying the results

De-identified subject-level data is provided as supporting files. High-resolution CFS scores are provided in S1 File, ES-based burden scores are provided in S2 File, and SVM-based burden scores are provided in S3 File.

### Summary statistics of detected PSH episodes

As previously noted, from the clinician-annotated case example, episode mean duration was 42 minutes, standard deviation was 16 minutes and duration ranged from 3 to 206 minutes. A description of the episode detection model-identified episodes from the entire dataset is provided in Table 3. The SVM model-based method detected a higher number of episodes in both PSH+ and PSH- patients compared to the ES-based method. Both methods detected a greater number of episodes and higher burden scores but similar mean episode durations in PSH+ patients compared with PSH- controls. However, when comparing the spread between the number of episodes detected in PSH+ and PSH- patients, the ES-based method saw a larger difference. This was quantified by calculating Cohen’s d for each method, which was 0.52 for the ES-based method and 0.44 for the SVM method. In contrast, the ES-based method generated a smaller difference in PSH burden compared to the SVM model-based method, with Cohen’s d of 0.30 and 0.36, respectively.

**Table 3:** Summary of detected PSH episodes for the first 14 days.

	ES-based method			SVM model-based method		
	PSH+ (n = 107)	PSH- (n = 114)	Effect size	PSH+ (n = 107)	PSH- (n = 114)	Effect size
Mean Number of Episodes (SD)	95.97 (68.30)	63.05 (57.39)	0.52	100.58 (36.87)	82.87 (43.82)	0.44
Mean Episode Duration (SD)	45.27 (181.53)	47.43 (199.83)	0.01	69.16 (181.31)	63.81 (135.78)	0.03
Mean Burden Score (SD)	0.23 (0.19)	0.17 (0.20)	0.30	0.36 (0.19)	0.29 (0.20)	0.36

Characteristics of detected PSH episodes and burden score considering the first 14 days of hospitalization for critically ill TBI patients, stratified by clinical diagnosis of PSH. Effect size reported as Cohen’s D. SVM: support vector machine; PSH: paroxysmal sympathetic hyperactivity; SD: standard deviation.

### PSH quantification trends

**Fig 4:**
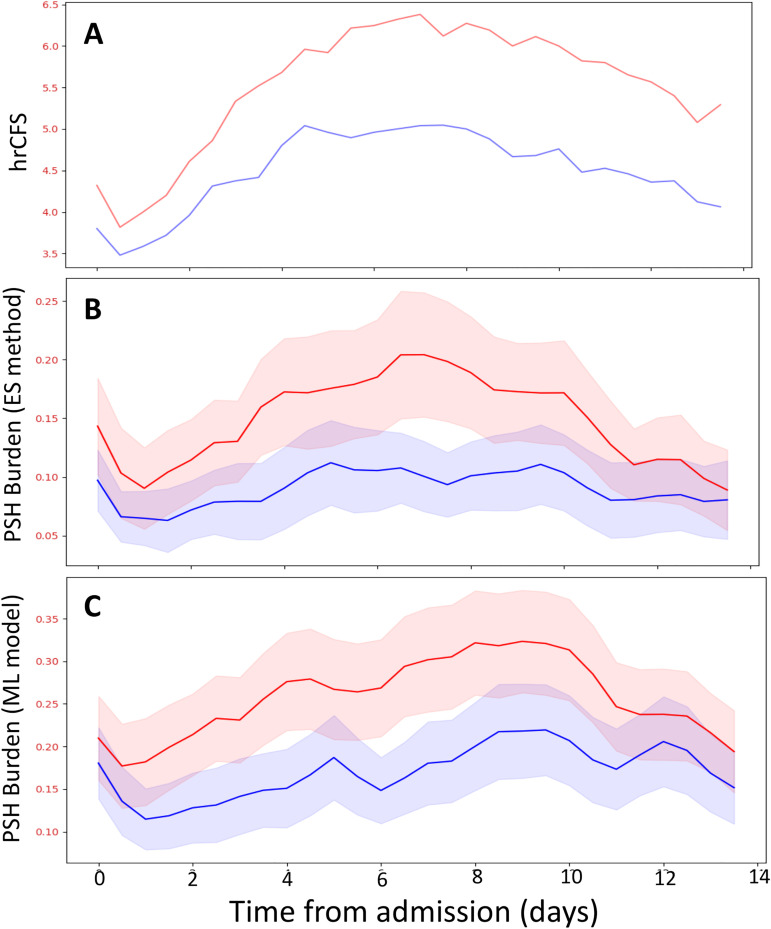
Face validity of automatic PSH quantification methods to distinguish between clinically defined PSH cases and controls. The high-resolution Clinical Feature Scale (hrCFS, 4A) and aggregate burden score trends from the expert system (ES) method (4B) and the machine learning (ML) model (4C) during the first 14 days of hospitalization are displayed, stratified by PSH clinical diagnosis. Score trends are computed from a series of 12-hour non-overlapping windows over the course of 14 days. Mean scores for PSH cases and controls are displayed as solid red and blue lines, respectively, and shaded areas represent 95% confidence intervals.

In Fig 4, we present the hrCFS and aggregate burden score trends during the first 14 days of hospitalization, stratified by PSH clinical diagnosis. Both the burden score and the hrCFS score trends are computed from a series of 12-hour non-overlapping windows over the course of 14 days, which amounts to 28 data points per series.

### Precision and recall

Table 4 summarizes the precision and recall computed from PSH episodes detected using the ES-based and SVM model-based methods, validated against the 180 human-annotated PSH episodes. While there is no significant difference in precision between the two methods, the model-based method shows a slight advantage in recall over the rule-based method.

**Table 4:** Episode detection method performance.

	Precision	Recall
ES-based method	0.518	0.649
SVM model-based Method	0.602	0.595

Precision and recall computed from PSH episode detected using expert system (ES)-based method and support vector machine (SVM) model-based method, when validated against human-annotated PSH episodes.

**Fig 5:**
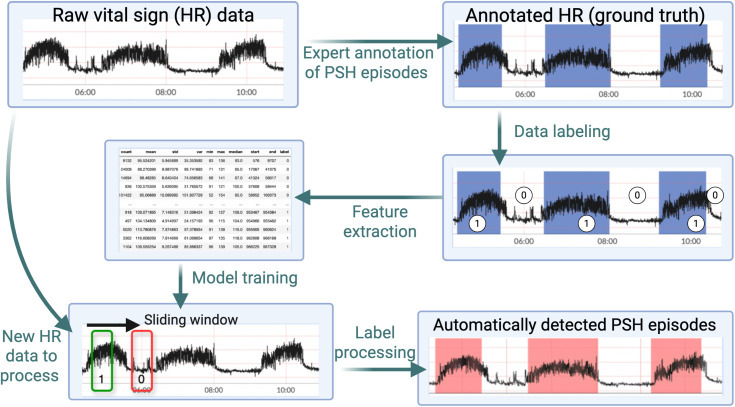
Illustrations of episode detection method performance. Examples of detected episodes (highlighted in yellow) and clinician’s annotations (highlighted in gray) overlayed onto heart rate, in beats per minute (bpm) tracings. 5A illustrates good performance with only small timing differences between annotated and detected episodes, and the majority of episodes are overlapping. 5B provides an example of a low-precision period, defined as detected episodes that were not annotated by the clinician (false positives). 5C illustrates a low-recall scenario, where clinician-annotated episodes were not detected by our methods (false negatives).

Fig 5 provides examples of detected episodes compared with human annotation, including heart rate recordings during epochs of high precision and recall (5A), low precision (5B), and low recall (5C).

## Discussion

In this study we demonstrate three automatic continuous VS-derived methods for quantifying PSH in critically ill acute TBI patients. These methods demonstrate initial face validity by distinguishing clinically identified PSH cases from controls in a dynamic manner that mimics the manual standard PSH quantification tool, the PSH-AM [1,2].

This study has several interesting findings. There is a difference in PSH automated quantifications between TBI patients clinically diagnosed with PSH and controls around one week post-injury, as demonstrated by non-overlapping confidence intervals in Fig 5B and 5C. This is the period when PSH is typically recognized clinically [1]. In addition, PSH burden among cases becomes more similar to controls towards the end of the two-week period (Fig 5B and 5C). This may correspond to spontaneous improvement in symptoms as part of the natural history of PSH (as many patients manifest only transient sympathetic hyperactivity after brain injury [15]), or to effective medical management. The qualitative differences in PSH episode burden trajectories compared to hrCFS trajectories suggests that these different methods could provide different information regarding sympathetic activity in the context of TBI. The episode detection-based burden scores aim to capture anomalous discrete periods of VS elevations from baseline, whereas the hrCFS is simply the sum of VS derangements at any given time, irrespective of time points before and after.

A variety of computational measurements can be gathered from calculated hrCFS and PSH episode detection tools with potential clinical relevance. Beyond the PSH episode burden, the duration, frequency, and amplitude of episodes all may have clinical relevance and could be tracked to determine efficacy of specific interventions. Additionally, a modified tool that combines both the hrCFS and episode detection methods might improve accuracy and validity and will be explored in future work.

**Fig 6:**
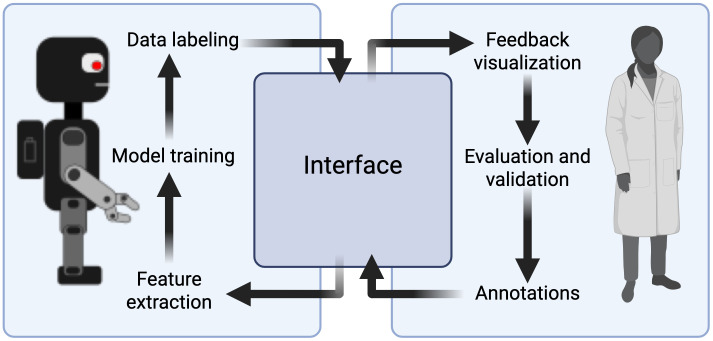
Framework for Human-Machine collaborative annotation and modeling. The above figure illustrates our envisioned human-in-the-loop method for iterative improvement of our PSH episode detection tools.

While precision and recall offer quantitative measures of method performance, they also highlight areas for improvement in both human and machine annotations. As seen in the examples of false positives and false negatives in Fig 6, human experts may sometimes overlook PSH episodes, and machine detection methods still need refinement to enhance accuracy. Developing an automated framework that iteratively learns from human annotations and continually refines its detection model could lead to more optimal results.

A challenge for this field is in defining the gold standard for PSH detection. The PSH-AM has emerged as the current gold standard but relies on phenomenology, subjective clinical judgements, and diagnoses of exclusion. It is the authors’ belief that post-TBI autonomic endophenotypes such as PSH could be better defined by data driven cluster analyses to identify biologically similar groups with similar physiologic expressions of autonomic function.

Presently, this work suggests proof of concept for automated PSH quantification but has several limitations. First, data from a single patient was used as ground truth for episode detection models. Therefor, these models may be overly constrained by this patient’s unique physiology and insensitive to detect PSH episodes across all patients. Individual differences in demographic features, brain injury patterns, comorbidities, and pharmacotherapy may affect both baseline physiologic parameters and the physiologic expression of PSH. Because an optimized PSH detection and quantification tool should be applicable to a diverse patient population, it will require refinement with robust training data from a similarly diverse patient sample. Additionally, rule-based constraints that leverage literature descriptions of classic PSH episodes (emphasizing simultaneity, recurrence, and duration of symptoms, for example) can also be incorporated. This approach may limit data-driven episode detection models to clinical expectations and avoid over-fitting to individual patient training data. The fact that we already see separation in case-control PSH quantifications using training data from a single patient is promising for future iterations.

The availability of physiologic data streams limits automated approximations of the PSH-AM, the current gold standard manual tool for PSH quantification. Specifically, we utilize only 3 of the 6 criteria comprising the Clinical Feature Scale (CFS, Table 1), to derive the hrCFS (heart rate, systolic blood pressure, and respiratory rate, but not temperature, sweating, or motor posturing). This could reduce sensitivity and specificity for identifying sympathetic hyperactivity. Additional sensors that could measure sweating and posturing (such as electrodermal activity sensors, sweat biosensors, and motion detectors) could improve performance but could also reduce feasibility and increase cost of applying these methods in real-world scenarios by requiring supplementary devices and infrastructure beyond standard clinical monitors. Unlike the high-resolution sampling of heart rate, respiratory rate, and blood pressure in our data, temperature measurements were sparse and inconsistent. The discrepancy in data granularity meant that imputation or weighting approaches to incorporate temperature into our episode detection models would not perform well. Our future work will explore incorporating temperature measurements and other sparsely collected data into a broader decision-support framework.

This study is the first attempt to automatically quantify PSH episodes beyond simple threshold-based approaches, and to mitigate the limitation of manual CFS computing. Herein, we utilized two AI methodologies to detect episodes of PSH, a more fundamental expert system based on detection rules, and a machine learning support vector machine model. These models were fine-tuned in an exceptionally data-constrained regime, using a single patient’s annotated PSH episodes. Despite this limitation, both models showed greater ability to identify PSH regions in a PSH+ cohort relative to those without the disease. As we upscale training data, we will combine elements of expert system and machine learning models to optimize performance within a novel, efficient human-in-the-loop anomaly detection system (Fig 6). This system will leverage human expertise and extracted information from published and peer-reviewed material to enhance machine learning models – a hybrid AI approach. We aim to develop a user interface allowing clinicians to not only navigate and annotate events from VS data, but also modify the detection rules. Based on feedback from clinicians, the machine can effectively learn patterns and iteratively refine rules to label the data. The ultimate goal is to have a tightly coupled and transparent system between clinicians and machines in which we can properly translate domain knowledge to machine language to identify plausible PSH episodes.

## Conclusions

This research presents methods for automatically quantifying PSH using patients’ high-resolution continuous VS data. Similar to prior work using manually derived daily CFS scores, our VS-based hrCFS differs across clinically defined PSH cases and controls during the time period when PSH is most often first diagnosed. Our episode detection methods successfully identify PSH episodes, which occur more frequently in patients with eventual clinical diagnoses of PSH. To address the limitations of our current study, future work will incorporate a human-in-the-loop framework, which we anticipate will enhance the performance of our detection methods by adaptive learning capabilities. By scaling up this process to include more individual patient data and by iteratively refining models using human feedback and insight, the performance and reliability of our automated PSH detection and quantification tools will continue to improve and may one day be implemented as real-time clinical decision support tools.

## Supporting information

S1 File High resolution clinical feature scale (hrCFS) scores. De-identified patient-level data used to generate Fig 4A.
(CSV)

S2 File Expert system-based episode detection burden scores. De-identified patient-level data used to generate Fig 4B.
(CSV)

S3 File Support vector machine (SVM) model-based episode detection burden scores. De-identified patient-level data used to generate Fig 4C.
(CSV)
